# Allergen immunotherapy for allergic rhinoconjunctivitis: a systematic overview of systematic reviews

**DOI:** 10.1186/s13601-017-0159-6

**Published:** 2017-08-08

**Authors:** Ulugbek Nurmatov, Sangeeta Dhami, Stefania Arasi, Graham Roberts, Oliver Pfaar, Antonella Muraro, Ignacio J. Ansotegui, Moises Calderon, Cemal Cingi, Stephen Durham, Roy Gerth van Wijk, Susanne Halken, Eckard Hamelmann, Peter Hellings, Lars Jacobsen, Edward Knol, Desiree Larenas-Linnemann, Sandra Y. Lin, Vivian Maggina, Hanneke Oude-Elberink, Giovanni Pajno, Ruby Panwankar, Elideanna Pastorello, Constantinos Pitsios, Giuseppina Rotiroti, Frans Timmermans, Olympia Tsilochristou, Eva-Maria Varga, Jamie Wilkinson, Andrew Williams, Margitta Worm, Luo Zhang, Aziz Sheikh

**Affiliations:** 1 Division of Population Medicine, School of Medicine, Cardiff University, Wales, UK; 2 Evidence-Based Health Care Ltd, Edinburgh, UK; 3 Allergy Unit - Department of Pediatrics, University of Messina, Via Consolare Valeria - Gazzi, Messina, Italy; 4 Molecular Allergology and Immunomodulation-Department of Pediatric Pneumology and Immunology, Charité Medical University, Augustenburger Platz 1, Berlin, Germany; 5 The David Hide Asthma and Allergy Research Centre, St Mary’s Hospital, Newport Isle of Wight, UK; 6 NIHR Biomedical Research Centre, University Hospital Southampton NHS Foundation Trust and Faculty of Medicine, University of Southampton, Southampton, UK; 7 Department of Otorhinolaryngology, Head and Neck Surgery, Universitätsmedizin Mannheim, Medical Faculty Mannheim, Heidelberg University, Mannheim, Germany; 8 Center for Rhinology and Allergology, Wiesbaden, Germany; 9 Food Allergy Referral Centre Veneto Region, Department of Women and Child Health, Padua General University Hospital, Padua, Italy; 10 Hospital Quiron Bizkair, Bilbao, Spain; 11 National Heart and Lung Institute, Imperial College, London, UK; 12 Department of ENT, Eskisehir Osmangazi University Medical Faculty, Eskisehir, Turkey; 13 Section of Allergology, Department of Internal Medicine, Erasmus MC, Rotterdam, The Netherlands; 14 Hans Christian Andersen Children’s Hospital, Odense University Hospital, Odense, Denmark; 15 Children’s Center Bethel, EvKB, Bieledelf and Allergy Center, Buhr-University, Bochum, Germany; 16 Laboratory of Experimental Immunology, University Hospitals Leuven, Louvain, Belgium; 17 ALCAllergy Learning and Consulting, Copenhagen, Denmark; 18 University Medical Center, Utrecht, The Netherlands; 19 Hospital Medica Sur, Mexico City, Mexico; 20 Department of Otolaryngology-Head & Neck Surgery, John Hopkins, Baltimore, MD USA; 21 Allergy and Clinical Immunology Unit, 2nd Department of Pediatrics, University of Athens, P&A Kiriakou Children’s Hospital, Athens, Greece; 22 Department of Allergology, Groningen Research Institute for Asthma and COPD (GRIAC), University Medical Center Groningen, University of Groningen, Groningen, The Netherlands; 23 Department of Pediatrics, Nippon Medical School, Tokyo, Japan; 24 University of Milano, Milan, Italy; 25 Medical School, University of Cyprus, Nicosia, Cyprus; 26 The Royal National Throat, Nose and Ear Hospital, University College London, London, UK; 27 Netherlands Anafylaxis Network, Dordrecht, The Netherlands; 28 Department of Paediatrics, Respiratory and Allergic Disease Division, Medical University Graz, Graz, Austria; 29 Pharmaceutical Group of the European Union, Brussels, Belgium; 30 Guy’s and St Thomas’ NHS Foundation Trust, London, UK; 31 Charitie-Universitatsmedizin, Berlin, Germany; 32 Beijing Institute of Otolaryngology, Beijing, China; 33 Allergy and Respiratory Research Group, The University of Edinburgh, Edinburgh, UK

**Keywords:** Allergy, Allergen immunotherapy, Allergic rhinitis, Allergic rhinoconjuctivitis, Hay fever, Rhinitis

## Abstract

**Background:**

The European Academy of Allergy and Clinical Immunology (EAACI) is developing Guidelines on Allergen Immunotherapy (AIT) for Allergic Rhinoconjunctivitis (ARC). To inform the development of recommendations, we sought to critically assess the systematic review evidence on the effectiveness, safety and cost-effectiveness of AIT for ARC.

**Methods:**

We undertook a systematic overview, which involved searching nine international biomedical databases from inception to October 31, 2015. Studies were independently screened by two reviewers against pre-defined eligibility criteria and critically appraised using the Critical Appraisal Skills Programme (CASP) Systematic Review Checklist for systematic reviews. Data were descriptively synthesized.

**Results:**

Our searches yielded a total of 5932 potentially eligible studies, from which 17 systematic reviews met our inclusion criteria. Eight of these were judged to be of high, five moderate and three low quality. These reviews suggested that, in carefully selected patients, subcutaneous (SCIT) and sublingual (SLIT) immunotherapy resulted in significant reductions in symptom scores and medication requirements. Serious adverse outcomes were rare for both SCIT and SLIT. Two systematic reviews reported some evidence of potential cost savings associated with use of SCIT and SLIT.

**Conclusions:**

We found moderate-to-strong evidence that SCIT and SLIT can, in appropriately selected patients, reduce symptoms and medication requirements in patients with ARC with reassuring safety data. This evidence does however need to be interpreted with caution, particularly given the heterogeneity in the populations, allergens and protocols studied. There is a lack of data on the relative effectiveness, cost-effectiveness and safety of SCIT and SLIT. We are now systematically reviewing all the primary studies, including recent evidence that has not been incorporated into the published systematic reviews.

**Electronic supplementary material:**

The online version of this article (doi:10.1186/s13601-017-0159-6) contains supplementary material, which is available to authorized users.

## Background

Allergic rhinoconjunctivitis (ARC) is a very common chronic condition that can result in considerable morbidity and impairment in quality of life [1–3]. The disease is triggered by exposure to seasonal and/or perennial allergens and, depending on the nature of the allergenic trigger(s) and patterns of exposure, symptoms may be intermittent, persistent or persistent with intermittent exacerbations [4]. Allergic rhinitis (AR) is typically characterized by symptoms of nasal obstruction, a watery nasal discharge, sneezing and itching; there is also often involvement of the conjunctiva, which manifests with itching, injection of the conjunctiva and tearing [5]. In addition, there may be an impact on the ability to concentrate, an adverse impact on school and work performance [6, 7], and interference with daily activities and sleep; allergic rhinitis is a risk factor for the development of asthma [8].

In most cases, symptoms can be controlled with attempts to avoid the allergenic trigger and pharmacotherapy, including oral, intranasal and H_1_-antihistamine eye drops, intranasal corticosteroids and anti-leukotrienes; these agents can be used as monotherapy or in combination [4, 9, 10]. Allergen immunotherapy (AIT) is an additional treatment option for those with troublesome disease that remains inadequately controlled by other therapies [11–13]; it has also been shown to have a disease modifying effect [14]. The problem of uncontrolled ARC, despite maximum medical treatment, continues to represent a therapeutic challenge in some patients [15].

We are undertaking a comprehensive, systematic synthesis of the evidence in relation to AIT for ARC to inform new European Academy of Allergy and Clinical Immunology (EAACI) Guidelines on Allergen Immunotherapy (AIT) for ARC. Due to the volume of primary trial data available for ARC, we have divided the evidence synthesis process into an initial systematic overview of the previous published systematic reviews. This will be followed by a second review focusing on the primary studies. This initial paper aims to provide a rigorous overview of current systematic review evidence on the effectiveness, safety and cost-effectiveness of AIT [16]. A second publication will focus on a systematic review of the primary publications.

## Methods

This systematic overview of systematic reviews was conducted and reported in accordance with the Preferred Reporting Items for Systematic Reviews and Meta-Analysis (PRISMA) guidelines (Additional file 1: Appendix 3). Our protocol is registered with the PROSPERO International Prospective Register of Systematic Reviews (CRD42016035373), which is reported in full in Clinical and Translational Allergy [17]. We therefore confine ourselves here to providing a synopsis of the methods employed.

### Search strategy

A highly sensitive search strategy was developed and validated study design filters were applied to retrieve articles pertaining to the use of AIT for ARC from electronic bibliographic databases. We used the systematic review filter developed at McMaster University Health Information Research Unit (HIRU) [18].

We searched the following databases: Cochrane Library including, Cochrane Database of Systematic Reviews (CDSR), Database of Reviews of Effectiveness (DARE), CENTRAL (Trials), Methods Studies, Health Technology Assessments (HTA), Economic Evaluations Database (EED), MEDLINE (OVID), Embase (OVID), CINAHL (Ebscohost), ISI Web of Science (Thomson Web of Knowledge), TRIP Database (http://www.tripdatabase.com).

The search strategy was developed on OVID MEDLINE and then adapted for the other databases (see Additional file 1). In all cases, the databases were searched from inception to October 31, 2015. Additional references were located through searching the references cited by the identified studies, and unpublished work, while research in progress was identified through discussion with experts in the field. There were no language restrictions employed; where possible, relevant literature was translated into English.

### Inclusion criteria

#### Patient characteristics

We focused on systematic reviews of studies conducted on patients of any age with a physician-confirmed diagnosis of ARC or AR, plus evidence of clinically relevant allergic sensitization (e.g., skin prick test or specific-IgE).

#### Interventions of interest and comparator

We were interested in AIT for relevant allergens in ARC (e.g. pollen, house dust mites, animal dander, cockroach and molds), including modified allergens. These could have been administered through any route (e.g. subcutaneous (SCIT), sublingual (SLIT), oral (OIT), intranasal (LNIT), epicutaneous, intradermal or intra-lymphatic) compared with placebo or any active comparator.

#### Study designs

We were interested in evidence from systematic reviews.

#### Study outcomes

The primary outcome of interest was measures of short-term (i.e. during treatment) and long-term (i.e. at least a year after discontinuation of AIT) measures of effectiveness assessed by symptom and/or medication scores [16].

Secondary outcomes of interest included: assessment of disease specific quality of life; threshold of allergen exposure to trigger symptoms in an environmental exposure chamber or allergen challenge; safety as assessed by local and systemic reactions in accordance with the World Allergy Organization’s grading system of side effects [19, 20]; and health economic analyses from the perspective of the health system/payer.

### Study selection

All references were uploaded into the systematic review software DistillerSR and underwent initial de-duplication. Study titles were independently checked by two reviewers (UN and SD) according to the above selection criteria and categorized as: included, not included or unsure. For those papers in the unsure category, abstracts were retrieved and re-categorized as above. Any discrepancies were resolved through discussion and, if necessary, a third reviewer was consulted (AS). Full text copies of potentially relevant studies were obtained and their eligibility for inclusion independently assessed. Studies that did not fulfil all of the inclusion criteria were excluded.

### Quality assessment strategy

Quality assessments were independently carried out on each systematic review by two reviewers (UN and SA) using the relevant version of the Critical Appraisal Skills Programme (CASP) quality assessment tool for systematic reviews [21]. Any discrepancies were resolved by discussion or, when agreement could not be reached, arbitration by a third reviewer (SD).

### Data extraction, analysis and synthesis

Data were independently extracted onto a customized data extraction sheet in DistillerSR by two reviewers (UN and SA), and any discrepancies were resolved by discussion or, if agreement could not be reached, by arbitration by a third reviewer (SD). We produced a descriptive summary with data tables to support a narrative synthesis of the data.

## Results

### Characteristics of included systematic reviews

**Fig. 1:**
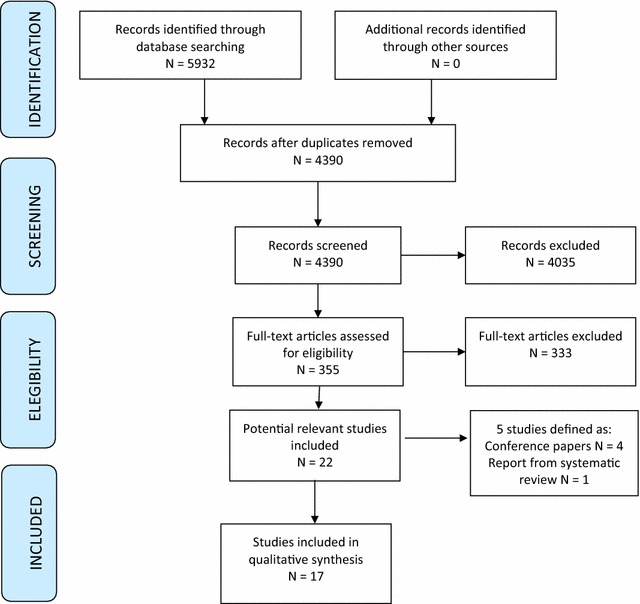
PRISMA flow diagram

Our searches yielded a total of 5932 potentially eligible systematic reviews and primary studies. Twenty-two potential systematic reviews were identified; five of these were however conference papers (n = 4) and a report on a systematic review (n = 1). Seventeen reviews met our inclusion criteria (see PRISMA flow diagram, Fig. 1). We were unable to obtain a translation for one of the reviews [30].

These 17 systematic reviews included synthesis of data from 466 randomized controlled trials (RCTs), of which ~300 were unique (we were unable to be more specific because not all of these systematic reviews provided a comprehensive list of included studies; see Additional file 2: Table S1). There were four systematic reviews investigating SCIT [22–25], eight SLIT [26–33], four both SCIT and SLIT [34–37], and one investigating several different routes of administration of AIT including SCIT, SLIT, OIT and LNIT [38].

The majority of systematic reviews were led by teams from the UK (n = 5) [25, 29, 31, 34, 36], followed by the USA (n = 4) [22, 24, 28, 35], Italy (n = 3) [26, 27, 32], the Netherlands (n = 2) [30, 38], China (n = 2) [23, 33], and Canada (n = 1) [37]. Twelve systematic reviews included participants of any age (i.e. children and adults) [22–29, 31, 34, 36, 37], four included children aged up to 18 years of age [32, 33, 35, 38].

In nine of the systematic reviews, data were pooled and the results of meta-analyses were provided (see Table 1) [22, 25–27, 29, 31, 33, 34, 37].

**Table 1:** Characteristics of included studies

References	Title	Countrys	Number of studies included (number of participants)	Type of immunotherapy (intervention vs. comparator)	Type of allergen/AIT protocol	Timeframe over which evaluation undertaken	Authors’ results & conclusions	Risk of bias
Calderon et al. [25]	Allergen injection immunotherapy for seasonal allergic rhinitis	UK	51 (2871: 1645 verum; 1226 placebo)	SCIT versus placebo	Pollen/continuous AIT	Up to February 2006	SCIT is a safe and valid treatment option in pts (children and adults) with SAR. MAs showed an overall reduction in SS (SMD −0.73 (95% CI −0.97 to −0.50, P < 0.00001) and MS (SMD of −0.57 (95% CI −0.82 to −0.33, P < 0.00001) in the IT group. Clinical interpretation of the effect size is difficult. Adrenaline was given in 0.13% (19 of 14,085 injections) of those on IT and in 0.01% (1 of 8278 injections) of the placebo group for treatment of AEs. There were no fatalities	Low
Di Bona et al. [27]	Efficacy of grass pollen allergen sublingual immunotherapy tablets for seasonal allergic rhinoconjunctivitis: a systematic review and meta-analysis	Italy	13 (4659)	SLIT (only tablets) versus placebo	Grass pollen/cluster AIT	Up to April 2014	There is small benefit in active group in reducing the SS (SMD, −0.28; 95% CI, − 0.37 to −0.19; P < .001) and the MS (SMD, −0.24; 95% CI, −0.31 to −0.17; P < .001) in SAR pts. The magnitude of benefits is lower in children. Also, safety data are not encouraging (7 pts in the SLIT group reported severe treatment-related AEs requiring adrenaline)	Moderate
Di Bona et al. [26]	Efficacy of sublingual immunotherapy with grass allergens for seasonal allergic rhinitis: a systematic review and meta-analysis	Italy	19 (2971)	SLIT versus placebo	Grass pollen/pre-coseasonal and continuous AIT	Up to January 2010	SLIT with grass allergens is effective in significantly reducing both SS (SMD, –0.32; 95% CI, –0.44 to –0.21; P < .0001) and MS (SMD, –0.33; 95% CI, –0.50 to –0.16; P < .0001) compared to placebo. However, the magnitude of effectiveness is low. Sub-analyses show major magnitude of effectiveness in adult’s versus children. A course of treatment ≤12 wks with a monthly allergen dose of 450 mcg seems to be the best treatment option	Moderate
Dranitsaris et al. [37]	Sublingual or subcutaneous immunotherapy for seasonal allergic rhinitis: an indirect analysis of efficacy, safety and cost	Canada	20 (6405)	SLIT (tablets: Oralair/Grazax) versus placebo compared with SCIT versus placebo	Grass/pre-coseasonal and continuous AIT	Up to December 2012	The indirect analysis suggests improved efficacy in AR symptom control with Oralair™ (SMD, −0.21; P = 0.007) and Grazax™ (SMD, −0.18; P = 0.018) over SCIT and comparable safety. In Canada, Oralair™ is associated with cost savings against year-round SCIT ($2471), seasonal SCIT ($948) and Grazax™ ($1168) during the first year of therapy	High
Dretzke et al. [36]	Subcutaneous and sublingual immunotherapy for seasonal allergic rhinitis: a systematic review and indirect comparison	UK	SCIT versus placebo: 17 RCTs; SLIT versus placebo: 11 RCTs; SCIT versus SLIT: 1 RCT	SCIT and SLIT versus placebo and SCIT versus SLIT	Pollen, mold/heterogeneous protocols	August 2009 to April 2011	SCIT and SLIT are effective versus placebo (strength of effectiveness higher in adults than in children) in improving SS [(SCIT: SMD, 20.65; 95% CI, 20.85 to 20.45; P < .00001); (SLIT: SMD, 20.33; 95% CI, 20.42 to 20.25; P < .00001)]; MS [(SCIT: SMD, 20.55; 95% CI, 20.75 to 20.34; P < .00001); (SLIT: SMD, 20.27; 95% CI, 20.37 to 20.17; P < .00001)]; HR-QoL. The superiority of effectiveness of one route of administration over the other cannot be consistently demonstrated	Low
Erekosima et al. [22]	Effectiveness of subcutaneous immunotherapy for allergic rhinoconjunctivitis and asthma: a systematic review	USA	61 (3577): 12 AA, 23 AR, and 26 combined AA & AR RCTS	SCIT versus placebo/SCIT versus pharmacotherapy/SCIT versus SCIT (different regimens)	Pollen, HDM, mold, animal dander/heterogeneous protocols	1967 to May 2012	Moderate to strong evidence supports the effectiveness of SCIT for treatment of adult pts with AR and/or AA, particularly with single-allergen IT regimens. AEs to SCIT are common, but no deaths are reported in the included studies	High
Feng et al. [23]	Cluster subcutaneous allergen specific immunotherapy for the treatment of allergic rhinitis	China	8 (567)	Cluster SCIT versus placebo/cluster SCIT versus conventional SCIT	Pollen, HDM, animal dander/heterogeneous protocols	1980 to May 2013	Though cluster SCIT is safe, because of limited evidence authors could not conclude affirmatively that cluster SCIT is an effective option (in terms of reduction of SS and MS) for the treatment of patients with ARs	Moderate
Hoeks et al. [30] (Dutch study translation not possible)	Sublingual immunotherapy in children with asthma or rhinoconjunctivitis: not enough evidence because of poor quality of the studies; a systematic review of literature							
Kim et al. [35]	Allergen-specific immunotherapy for pediatric asthma and rhinoconjunctivitis: a systematic review	USA	SCIT versus placebo: 13 RCTs (920); SLIT versus placebo: 18 RCTs (1583); SCIT versus SLIT: 3 RCTs (135)	SCIT versus placebo/SLIT (only aqueous formulation) versus placebo/SCIT versus SLIT (only aqueous formulation)	Pollen, HDM, mold/heterogeneous protocols	Up to May 2012	Both SCIT and SLIT are effective for the treatment of AA and AR in children. The strength of evidence is moderate that SCIT improves AA and AR SS and low that SCIT improves AA MS. Strength of evidence is high that SLIT improves AA SS and moderate that SLIT improves AR SS and AR MS. The evidence is low to support SCIT over SLIT for improving AA and AR SS or MS	Moderate
Lin et al. [28]	Sublingual immunotherapy for the treatment of allergic rhinoconjunctivitis and asthma: a systematic review	USA	63 (5131): SLIT versus placebo 46 RCTs; SLIT versus another SLIT protocol without a placebo group 9 RCTs; SLIT versus ST without placebo 8 RCTs	SLIT versus placebo/SLIT versus ST/SLIT versus SLIT (different regimens)	Pollen, HDM, mold/heterogeneous protocols	Up to December 2012	There is moderate grade level of evidence to support the effectiveness of SLIT for AR and AA in adults and children. No life-threatening AEs were noted in this review	High
Meadows et al. [34]	A systematic review and economic evaluation of subcutaneous and sublingual allergen immunotherapy in adults and children with seasonal allergic rhinitis	UK	SCIT versus placebo: 17 RCTs; SLIT versus placebo:11 RCTs; SCIT versus SLIT:16 RCTs	SCIT versus placebo/SLIT versus placebo/SCIT versus SLIT	Pollen/conventional protocol	Up to April 2011	Effectiveness (SS, MS, HR-QoL) of both SCIT and SLIT versus placebo has been demonstrated in adults with SAR +/− seasonal AA. There is less evidence for children, but some results in favour of SLIT were statistically significant. However, overall the extent of this effectiveness in terms of clinical benefit is unclear. Both SCIT and SLIT may be cost-effective compared with pharmacotherapy from around 6 years (threshold of £20,000–30,000 per QALY)	Low
Purkey et al. [24]	Subcutaneous immunotherapy for allergic rhinitis: an evidence based review of the recent literature with recommendations	USA	12 (1512)	SCIT versus placebo/SCIT versus SLIT	Pollen, HDM/heterogeneous protocols	From 2006 to 2011	SCIT improves SS, MS, SMS and HR-QoL. Authors recommend SCIT for pts with seasonal or perennial AR not responsive to conservative medical therapy, and whose symptoms significantly affect HR-QoL	High
Radulovic et al. [29]	Systematic reviews of sublingual immunotherapy (SLIT)	UK	60 RCTs in SR, 49 suitable for MA; Age: 34 RCTs in adults and 15 in children	SLIT versus placebo	Pollen, HDM, cat/heterogeneous protocols	Up to August 2009	SLIT is safe and effective in reducing AR- SS (SMD, −0.49; 95% CI −0.64 to −0.34, P < 0.00001) and -MS (SMD −0.32; 95% CI −0.43 to −0.21, P < 0.00001) compared with placebo. The magnitude of benefit appears to be major for SLIT to HDM. No difference of efficacy were found between children and adults. There was too much heterogeneity to evaluate differences between different sublingual preparations (drops vs. tablets) and doses and about HR-QoL	Low
Roder et al. [38]	Immunotherapy in children and adolescents with allergic rhinoconjunctivitis: a systematic review	The Netherlands	28 RCTs (1619): 6 SCIT, 4 LNIT, 7 OIT and 11 SLIT	SCIT/SLIT/LNIT/OIT versus placebo/ST/different administration forms of IT	Different pollen or HDM or mold/continuous or cluster protocol	Up to June 2006	There is at present insufficient evidence that IT in any administration form has a positive effect on symptoms and/or medication use in children and adolescents with AR	High
Sopo et al. [32]	Sublingual immunotherapy in asthma and rhinoconjunctivitis; systematic review of paediatric literature	Italy	8 RCTs	SLIT versus placebo	Pollen, HDM/conventional protocol	Up to June 2003	SLIT can be currently considered to have low to moderate clinical efficacy in children ≥4 yrs of age, monosensitised to HDM, and suffering from mild to moderate persistent AR. No clinically relevant results are shown, independently from statistical significance, in the use of SLIT for AA and AR due to seasonal allergens and for AR to HDM in children	High
Wilson et al. [31]	Sublingual immunotherapy for allergic rhinitis: systematic review and meta-analysis	UK	22 (979)	SLIT versus placebo/SLIT versus SCIT	Pollen, animal dander, HDM	Up to September 2002	SLIT is effective and safe. Overall there was a significant reduction in both SS (SMD −0.42, 95% CI −0.69 to −0.15; P = 0.002) and MS (SMD −0.43 95% CI −0.63 to −0.23; P = 0.00003) following SLIT. However, no significant benefit was found in those studies involving only children, though they had a sample size too small to be conclusive. There were no significant differences in benefit according to the allergen administered. Increasing duration of treatment does not clearly increase efficacy. The total dose of allergen administered may be important but insufficient data was available to analyse this factor	High
Zhang et al. [33]	Efficacy and safety of dust mite sublingual immunotherapy for pediatric allergic rhinitis: A meta-analysis	China	9 RCTs (663)	SLIT versus placebo	HDM	Up to May 2014	SLIT is effective and safe. There was no significant difference in improvement in children with allergic rhinitis nasal symptom score aspect [SMD = 0.06, 95% CI (−0.13, 0.25), P = 0.55]. However, the medication use in intervention group significantly decreased compared with placebo [SMD = −0.61, 95% CI (−0.94 to −0.27), P = 0.0004]	Moderate

*AA* allergic asthma, *AE* adverse event, *AR* allergic rhinitis, *HDM* house dust mite, *HR*-*QoL* health related quality of life, *IT* immunotherapy, *LNIT* nasal immunotherapy, *MA* meta-analysis, *MS* medication scores, *OIT* oral immunotherapy, *P P* value, *pt* patient, *QALY* quality-adjusted life-year, *RCT* randomized controlled trial, *SAR* seasonal allergic rhinitis, *SR* systematic review, *SCIT* subcutaneous immunotherapy, *SLIT* sublingual immunotherapy, *SS* symptom scores, *ST* standard treatment (anti-H_1_, …)

### Quality assessment of systematic reviews

Quality assessment of these systematic reviews is summarized in Table 2. Overall, the quality of included reviews were high to moderate, with only three studies being judged as being of low quality. Eight studies were considered at low risk of bias [23–25, 27, 29, 32, 34, 36], five studies were at moderate risk of bias [22, 26, 28, 33, 35], and three were judged as being at high risk of bias [31, 37, 38]. We then used a modified version of the World Health Organization’s Health Evidence Network system, as employed by Black et al. [39], for appraising evidence, which classifies evidence into strong, moderate or weak; this assessment being based on a combination of the overall consistency, quality, and volume of evidence uncovered (see Table 3).

**Table 2:** Critical appraisal of included systematic reviews (N = 17)

References	Focused question	Inclusion of appropriate studies	Inclusion of eligible studies	Quality assessment of studies	Appropriateness of synthesis	Overall results of review	Applicability to local populations	Considering all relevant outcomes	Benefits versus harms/costs	Overall quality assessment
Calderon et al. [25]	✓	✓	✓	✓	✓	✓	n/a	✓	✓	High
Di Bona et al. [27]	✓	✓	✓	✓	✓	✓	n/a	✓	✓	High
Di Bona et al. [26]	✓	✓	X	✓	✓	✓	n/a	✓	n/a	Moderate
Dranitsaris et al. [37]	✓	✓	Unclear	Unclear	✓	✓	n/a	✓	✓	Low
Dretzke et al. [36]	✓	✓	✓	✓	✓	✓	n/a	✓	✓	High
Erekosima et al. [22]	✓	✓	✗	✓	n/a	✓	n/a	✓	✓	Moderate
Feng et al. [23]	✓	✓	✓	✓	✓	✓	n/a	✓	✓	High
Hoeks et al. [30]										
Kim et al. [35]	✓	✓	✗	✓	✓	✓	n/a	✓	✓	Moderate
Lin et al. [28]	✓	✓	✗	✓	✓	✓	n/a	✓	✓	Moderate
Meadows et al. [34]	✓	✓	✓	✓	✓	✓	n/a	✓	✓	High
Purkey et al. [24]	✓	✓	✓	✓	✓	✓	n/a	✓	✓	High
Radulovic et al. [29]	✓	✓	✓	✓	✓	✓	n/a	✓	✓	High
Roder et al. [38]	✓	✓	Unclear	Unclear	✓	✓	n/a	✓	n/a	Low
Sopo et al. [32]	✓	✓	✓	✓	✓	✓	n/a	✓	n/a	High
Wilson et al. [31]	✓	✓	Unclear	Unclear	Unclear	✓	n/a	✓	✓	Low
Zhang et al.[33]	✓	✓	✓	Unclear	✓	✓	n/a	✓	n/a	Moderate

**Table 3:** Summary of evidence to support the effectiveness of AIT

Study ID	SLIT	SCIT
Calderon et al. [25]	NA	+++
Di Bona et al. [27]	+/−	NA
Di Bona et al. [26]	+/−	NA
Dranitsaris et al. [37]	++ (indirect analysis)	NA
Dretzke et al. [36]	+++	+++
Erekosima et al. [22]	NA	++/+++
Feng et al. [23]	NA	–
Kim et al. [35]	++	++
Lin et al. [28]	++	NA
Meadows et al. [34]	++ (only in adults)	++ (only in adults)
Purkey et al. [24]	NA	+++
Radulovic et al. [29]	++/+++	NA
Röder et al. [38] ^a^	–	–
Sopo et al. [32]	+/++	NA
Wilson et al. [31]	++ (only in adults)	NA
Zhang (2014)	++ (MS)/− (SS)	NA

Effectiveness is overall referred to symptom and medication scores unless otherwise stated

+++, Strong evidence; ++, Moderate evidence; +, Some/limited evidence; +/−, Unclear evidence; –, No evidence; SS, symptom scores; MS, medication scores

^a^Same results were referred also to oral immunotherapy and intranasal immunotherapy

### SCIT

#### Effectiveness of SCIT as assessed by symptom and medication scores

##### ARC symptom scores

There were four studies that evaluated the effectiveness of SCIT in children and adults [22–25]. The quality of evidence from included systematic reviews was high. Calderon et al., conducted a high quality Cochrane systematic review of SCIT for seasonal allergic rhinitis (SAR) covering studies up to 2006 [25]. Meta-analysis from 15 RCTs demonstrated a significant reduction in symptom score (SS) (SMD −0.73 (95% CI −0.97 to −0.50, P < 0.00001) in the intervention group when compared to placebo. The systematic review by Erokosima et al., judged to be of moderate quality, covered studies to 2012; it reported that 20 out of 23 included RCTs consistently showed greater SS improvement in the SCIT group than the comparator arm (usual care) [22]. Purkey et al., who analyzed data from 12 RCTs up to 2011 narratively, reported a significant decrease in allergic rhinitis symptom scores [24].

The high quality meta-analysis by Feng et al., which included eight cluster SCIT RCTs published up to 2013, however found that four trials demonstrated no significant difference in SSs compared to placebo: weighted mean difference (WMD) = −5.91 (95% CI −13.68 to 1.87; P = 0.14) [23].

Kim et al., evaluated three RCTs published up to 2012 with 285 pediatric patients with AR or ARC symptom scores and reported moderate strength evidence that SCIT controls AR or ARC symptoms better than placebo [35].

##### ARC medication scores

The systematic review and meta-analysis by Calderon et al. found that AIT significantly decreased medication scores (MS) with a SMD of −0.57 (95% CI −0.82 to −0.33, P < 0.00001) [25]. In the review by Erekosima et al., ten studies including 564 subjects found moderate evidence that SCIT decreased medication use in ARC [22]. However, combined symptom-medication scores (SMS) from six studies with 400 participants found only weak evidence to support that SCIT improves SMS. Feng et al. found no significant differences in MS between cluster SCIT versus placebo: combined WMD −1.27 (95% CI −2.83 to 0.29, P = 0.11) and WMD −0.01 (95% CI −0.16 to 0.13, P = 0.88), respectively [23]. Another high quality systematic review in this category by Purkey et al., in a descriptive analysis demonstrated that SCIT for AR significantly improved MS [24].

#### Effects of SCIT on secondary outcomes

##### Assessment of disease specific quality of life (QoL)

The review by Calderon et al. reported a clinically and statistically significant improvement in disease specific QoL in the immunotherapy group compared with placebo (SMD −0.52, 95% CI −0.69 to −0.34, P = 0.00001) [25]. Erekosima et al., who used the Rhinoconjunctivitis Quality of Life Questionnaire (RQLQ) and/or the Short Form 36 (SF36) questionnaire, also found high quality evidence to support the use of SCIT to improve disease-specific QoL (n = 539) for rhinitis/rhinoconjunctivitis [22]. The meta-analysis conducted by Feng et al., demonstrated that cluster SCIT was superior to placebo in improving overall QoL in two included studies (n = 104; WMD −0.79, 95% CI −1.10 to −0.47, P < 0.00001) [23]. Finally, Purkey et al., also found that, in four studies all of which used the RQLQ, that SCIT improved the QoL measure in patients with AR [24].

##### Threshold of allergen exposure to trigger symptoms in an environmental exposure chamber or allergen challenge

Two reviews investigated the impact on challenge tests [24, 25]. Calderon et al., reported an increase in the allergen provocation dose for the active treatment compared with placebo in 13 RCTs. 21 studies performed skin challenges and reported a reduction in the skin reactivity after SCIT [25]. However, in the more recent review, Purkey et al. reported conflicting results: one included RCT showed a reduction in immediate or delayed cutaneous responses grass pollen SCIT, two other RCTs also showed a reduction in symptoms in conjunctival provocation tests, but two other studies did not show any differences on either nasal provocation testing or skin reactivity to HDM results between active and placebo groups [24].

##### Safety

The Cochrane review by Calderon et al., demonstrated that SCIT had a low risk of severe adverse events. There were no fatalities in the included RCTs. Adrenaline (epinephrine) was given in 0.13% (19 of 14,085 injections) of those on SCIT and in 0.01% (1 of 8278 injections) of the placebo group for the treatment of adverse events (AEs) [25]. Erekosima et al. reported both local and systemic reactions: local reactions were common (5–58% participants, 3–10% injections); the most common systemic reactions were respiratory reactions (not broken down into upper or lower respiratory symptoms) (71% patients in the active group versus 88% in comparator group; up to 27% injections); there were 13 anaphylactic reactions in four RCTs and no fatalities were reported [22]. Purkey et al., reported that administering SCIT was safe in suitably selected patients and settings capable of responding to emergency situiations [24]. Feng et al., graded adverse events based on the European Academy of Allergology and Clinical immunology Position Paper: [40] no differences in local reactions between cluster SCIT and placebo (the combined risk difference (RD) 0.00, 95% CI −0.00 to 0.01, P = 0.40) with the same trend for systemic reactions (RD 0.00, 95% CI −0.00 to 0.01, P = 0.24) [24].

##### Comparative effectiveness of different AIT regimens

Two systematic reviews reported the comparison between different AIT regimens. Erekosima et al., assessed 23 SCIT RCTs, 20 of which showed a greater improvement in the SCIT group, two of these involved an active comparison: one compared SCIT with pharmacotherapy and the second trial compared with another unspecified control group [22]. Feng et al., also compared cluster SCIT versus conventional SCIT. There were no differences on SS or MS between cluster SCIT versus conventional SCIT: WMD 0.16, 95% CI −0.18 to 0.51; P = 0.36 and WMD −0.01, 95% CI −0.16 to 0.13, P = 0.88, respectively [23]. The incidence of local and systemic adverse reactions between cluster SCIT versus conventional SCIT also demonstrated no differences between these two groups (combined RR 1.13, 95% CI 0.63–2.03, P = 0.68, and RR 0.99, 95% CI 0.52–1.91, P = 0.98, respectively) [23].

##### Health economic analysis

There were no systematic reviews that reported on health economic outcomes.

### SLIT

#### Effectiveness of SLIT as assessed by symptom and medication scores

##### ARC symptom scores

Two systematic reviews and meta-analyses were conducted by Di Bona et al. In their first review (trials up to 2010) they reported that SLIT with grass pollen for SAR significantly reduced symptom scores (SMD −0.32, 95% CI −0.44 to −0.21, P < 0.0001) compared to placebo [26]. This was confirmed in their more recent (trials up to 2014) systematic review and meta-analyses in which they also reported a significant reduction symptom score in the active group compared with placebo (SMD −0.28, 95% CI −0.37 to −0.19, P < 0.01) [27]. This second review only included RCTs using SLIT in tablet form. Three other systematic reviews and meta-analyses also reported the impact of SLIT on AR or ARC symptom scores [29, 31, 33]. One meta-analysis (trials up to 2002) and a subsequent update (trials up to 2009) showed significant reductions in symptoms score in the SLIT group compared to placebo (SMD −0.42, 95% CI −0.69 to −0.15, P = 0.002 and SMD −0.49, 95% CI −0.64 to −0.34, P < 0.0001, respectively) [29, 31]. The third more recent (trials up to 2014) meta-analysis focusing just on children reported that there were no differences between intervention and placebo groups (SMD 0.06, 95% CI −0.13 to 0.25, P = 0.55) [33]. The other two reviews reported a narrative synthesis of RCTs: either moderate evidence that SLIT decreases AR or ARC symptoms, with nine of 36 included RCTs (up to 2012) reported greater than 40% improvement versus the comparator group [28] or no beneficial effect from SLIT in pediatric patients with AR in an older review (trials up to 2003) [32].

##### ARC medication scores

The two reviews by Di Bona et al. provided evidence that SLIT significantly reduced medication usage (SMD −0.33, 95% CI −0.50 to −0.16, P < 0.0001 and −0.24, 95% CI −0.31 to −0.17, P < 0.01, respectively) [26, 27]. A similar reduction in MS was seen in three other systematic reviews and meta-analyses (SMD −0.32, 95% CI −0.43 to −0.21, P < 0.00001 [29], SMD −0.43, 95% CI −0.63 to −0.23, P = 0.00003 [31], SMD −0.61, 95% CI −0.94 to −0.27, P = 0.0004 [33] compared with placebo). Lin et al., in a qualitative synthesis of RCTs, found that 38 of 41 studies (93%) found greater improvement in MS in the active group compared with the comparator group, with 16 studies demonstrating a strong effect [28].

#### Effects of SLIT on secondary outcomes

##### Assessment of disease specific QoL

Two systematic reviews assessed the effects of AIT on disease-specific QoL. Radulovic et al. found three studies that reported QoL, but assessments differed too much to allow them to include the data [29]. Lin et al. reported disease-specific QoL in eight studies involving 819 participants; seven of eight demonstrated a favorable change in the SLIT group compared with placebo [28].

##### Threshold of allergen exposure to trigger symptoms in an environmental exposure chamber or allergen challenge

One systematic review reported allergen sensitivity issues [31]. 13 RCTs measured cutaneous sensitivity and four studied nasal sensitivity. Seven studies reported no significant difference between active and placebo groups and in six studies, there was no comparison with placebo or relevant data presented.

##### Safety

Safety analysis of SLIT was reported in five systematic reviews [26–29, 31]; meta-analysis of data was reported in one of these systematic reviews [33]. Di Bona et al. reported a total of 4856 treatment-related AEs [3286 (2.6 AEs/patient) in the SLIT group and 1570 (1.34 adverse events/patient) in the placebo group]. The majority of adverse events were moderate; 3% in the SLIT group and 0.7% in the placebo group patients withdrew because of treatment-related adverse events [26]. The more recent review from the same research group demonstrated that adverse events were reported in 1384 of 2259 patients (61.3%) receiving SLIT and in 477 of 2279 patients (20.9%) receiving placebo. Withdrawal rate was higher in the SLIT group (6.0%) than in the placebo group (2.2%). No episodes of anaphylaxis were reported and seven patients required the use of adrenaline for systemic adverse events. (2) Lin et al. reported that local reactions were more frequent in the SLIT group (range 0.2–97%) than in the comparator groups (range 3–38.5%). There were no episodes of anaphylaxis or fatalities in any treated patients across studies [28].

The updated Cochrane review highlighted that the lack of a standardized grading system for reporting of AEs associated with SLIT made conducting meta-analysis impractical. None of the included RCTs reported severe systemic reactions, anaphylaxis or use of adrenaline [29]. Wilson et al., indicated that there were no systemic reactions in the RCTs. Minor local reactions, such as itching and swelling of the oral mucosa, were however reported almost in every included study [31]. The only systematic review and meta-analysis that pooled adverse events data quantitatively reported that there was no difference in the incidence of adverse events between active and placebo groups (OR 1.3, 95% CI 0.89–1.90, P = 0.17) [33].

##### Health economic analysis

There were no systematic reviews that reported on health economic outcomes.

### SCIT versus SLIT

There were four systematic reviews comparing SCIT and SLIT [34–37]; three of these also conducted indirect analysis of efficacy, safety and cost of SCIT versus SLIT [34, 36, 37]. The study by Dranitsaris et al. also employed an indirect analysis of efficacy, safety and cost of SLIT or SCIT for SAR [37].

#### Effectiveness as assessed by symptom and medication scores

##### ARC symptom scores

Dretzke et al. conducted a systematic review and indirect comparison (SCIT vs. SLIT) of included studies [36]. In studies where SCIT was compared with placebo, SCIT significantly decreased SS (SMD −0.65, 95% CI, −0.85 to −0.45, P < 0.00001. Indirect comparison based on one small low quality head-to-head RCT reported that standardized score difference for SS between SCIT versus SLIT was in favor of SCIT: 0.35, 95% CI 0.13–0.59. A HTA of SCIT and SLIT in adults and children with SAR demonstrated statistically significant effects of SCIT and SLIT compared with symptomatic treatment or placebo; of relevance, here however is that an indirect comparison suggested that SCIT was more effective than SLIT [34].

##### ARC medication scores

In an indirect comparison between SCIT and SLIT, the overall standardized score differences (SSDs) was 0.27 (95% CI 0.03–0.53) in favor of SCIT. SCIT also significantly reduced the combined symptom and medication score (SMS) (SMD −0.48 (95% CI −0.67 to −0.29, P < 0.00001)). Indirect comparison between SCIT and SLIT showed no difference in SMS between them (SSD 0.31, 95% CI −0.195.8 to 194.1) [26]. Kim et al., compared MS between SCIT and SLIT in children with asthma and ARC in 13 studies with 1078 participants. The strength of evidence was moderate that SLIT decreases medication use for the affected patients, but only low for SCIT [35]. A Health Technology Assessment (HTA) systematic review reported statistically significant results for SCIT and SLIT on MS [34]. An indirect comparison analysis between SCIT and SLIT found that SCIT was more beneficial for MS compared with SLIT, but this was associated with substantial residual heterogeneity of included studies.

##### Disease specific quality of life

Dretzke et al., reported that SCIT and SLIT improved disease specific QoL scores in patients with SAR when compared to controls (SMD −0.53, 95% CI −0.66 to −0.39, P < 0.00001 and SMD −0.37, 95% CI −0.52 to −0.22, P < 0.00001, respectively) [36]. There was however no differences in the impact on disease specific QoL scores between SCIT and SLIT trials (SSD 0.38, 95% CI −0.04 to 0.80). An HTA review reported beneficial effects of SCIT or SLIT on the QoL scores in patients with SAR compared with placebo; however, the indirect analysis could not find any difference on QoL scores between SCIT and SLIT [34].

#### Threshold of allergen exposure to trigger symptoms in an environmental exposure chamber or allergen challenge

There were no data to report for this outcome.

#### Safety

Dranitsaris et al. undertook an indirect comparison of safety between Oralair™, Grazax™ and SCIT [37]. The authors reported that there were no significant differences in the risk of discontinuation due to ARs between these three arms (Oralair™ 5.6% (95% CI 3.8–7.3); Grazax™ 3.5% (95% CI 1.7–5.2); and SCIT 2.7% (95% CI 1.3–4.2), respectively).

Dretzke et al. reported that 19% of systemic reactions were considered severe after SCIT treatment compared with only 2% of systemic reactions after SLIT. Discontinuation rates because of AEs were similar between SCIT and SLIT (approximately 3%) [36]. Kim et al. assessed safety outcomes for SCIT, SLIT and SCIT versus SLIT [35]. Safety of SCIT in children showed that local reactions were common, systemic reactions in 1–30% of patients, unspecified or general systemic reactions in 3–34% of patients, urticaria in 2–19% of patients. No anaphylactic reactions or death were reported. Safety data on SLIT in children showed that there were local reactions in 0.2–50% of patients in the SLIT group and 6–25% of patients receiving placebo. Systemic reactions were common, but no life-threatening allergic reactions were reported. One included study reported severe rhinitis and severe asthma symptoms in children who exceeded their maximum dose. Reducing the dosage of AIT resolved these reactions. Safety of SCIT versus SLIT showed that there were no systemic reactions in patients receiving SLIT; amongst 37 children receiving SCIT, however, four experienced systemic reactions (one anaphylaxis and three moderate to severe respiratory symptoms).

An HTA review reported that local reactions during SCIT and SLIT were common, but they resolved spontaneously without treatment [34]. Mild or moderate systemic reactions occurred in 4.4% of injections for SCIT. Nineteen percent of systemic reactions during SCIT treatment were considered to be severe, only 2% of systemic reactions following SLIT were graded as severe. Discontinuation due to AEs between these two types of AIT were similar (SCIT 3.0% and SLIT 3.4%). No fatalities were reported in any of these trials.

#### Health economic analysis

Two systematic reviews reported on health economic outcomes. Dranitsaris et al. reported that Oralair™ during the first year of AIT was associated with cost savings compared with yearly SCIT ($2471), seasonal SCIT ($948) and Grazax ($1168) [37]. Meadows et al. reported that where SCIT and SLIT were directly compared against each other, SCIT was found to be both more effective and more cost-effective over the long term [34]. The sample size of the only trial that directly compared the cost-effectiveness of SCIT and SLIT was, however, small (n = 64). They also calculated standard incremental cost-effectiveness ratios (ICERs), which demonstrated that both SCIT and SLIT were cost-effective at thresholds of £20,000 per quality-adjusted life-year (QALY). However, the included studies were conducted by sponsor organizations and there were some issues around transparency and/or robustness of parameters for most included studies.

### SCIT, SLIT, OIT or LNIT for children and adolescents

#### Effectiveness as assessed by symptom and medication scores

##### ARC symptom score

One systematic review by Roder et al., including studies up to 2006, evaluated four types of AIT—i.e. SCIT, SLIT, OIT and LNIT—in children and adolescents. This review included six SCIT, 11 SLIT, seven OIT and four LNIT RCTs. There was insufficient evidence that any of these AIT had positive impact on symptom scores of children or adolescents [38].

##### ARC medication score

There was insufficient evidence in the Roder et al. review to conclude if AIT delivered through these routes had a positive impact on the MS of children or adolescents [38].

#### Secondary outcomes

##### Safety

Local reactions were common; particularly in the intervention groups [38]. Systemic reactions were rare; only one SLIT trial reported an acute asthma exacerbation that required hospitalization, this occurring in the intervention group. However, another SLIT trial reported a serious AE in the placebo group. There were no anaphylactic reactions reported.

##### Assessment of disease specific quality of life, threshold of allergen exposure to trigger symptoms in an environmental exposure chamber, allergen challenge or health economic analysis

No data were available for these outcomes [38].

## Discussion

### Statement of principal findings

This comprehensive overview of the systematic review evidence has found that there is a substantive body of high quality evidence indicating that both SCIT and SLIT are effective in improving outcomes for patients with AR/ARC, although there are less positive efficacy data for children treated with SLIT. The safety profile of these treatment approaches seems acceptable, with a low risk of serious AEs if administered to appropriately selected patients and, particularly in relation to SLIT and for SCIT, if appropriate resuscitative facilities are available. There is limited evidence that these treatment options are likely to prove cost-effective. Less is known from systematic reviews about other routes of delivery of AIT. It is also difficult to draw any conclusions on the comparative effectiveness of SCIT versus SLIT versus other treatment routes.

### Strengths and limitations of this systematic review

We have undertaken a carefully conducted comprehensive overview of this substantial evidence base. We carefully identified relevant MeSH and keywords for AIT in patients with AR/ARC, and followed a detailed a priori protocol to minimize the risk of bias in our procedures. We also took care to ensure that those involved with undertaking relevant systematic reviews included in this overview were not directly involved in the assessment of their own studies.

The main limitations of this overview stems from the heterogeneity of populations studied, diversity of AIT regimens, allergen preparations, potency and dosage, and definitions of outcomes. There is also considerable overlap of primary studies included within these reviews, approximately a third of included studies are present in two or three of the reviews. Almost all the included systematic reviews reported issues to do with the diversity of the underpinning RCT evidence. There was, for example, considerable variability in scoring and reporting of primary and secondary outcomes including safety data, different allergen dosing and treatment schedules [22, 23, 35]. There was not only methodological diversity in the study design but also clinical diversity in the types of participants, their allergies, allergens treated, variety in dosing and treatment protocols, schedules, geographical treatment locations, quality, reporting and scoring of measured outcomes [22, 23, 28, 35]. As a result of the lack of a standardized grading system for reporting adverse events associated with AIT in included RCTs, these data could only be presented as descriptive data [29, 35]. These issues to do with diversity are compounded when synthesizing data at the systematic review level and care was therefore taken to ensure that we did not over-interpret findings from this initial overview of the literature.

Many of the limitations inherent in reviewing AIT relate to the changes in the therapeutic approach over the last five decades. While just crude allergen extracts were used in the early studies, more modern preparations are often combined with alum or an adjuvant such as monophosphoryl lipid A/AF or chemically modified into an allergoid. There has also been a move to better characterize AIT products to ensure they have a consistent and adequate allergen content. We cannot expect all to have similar efficacy characteristics. While the published systematic reviews incorporate this heterogeneity, they do not include the large number of recent RCTs assessing potent grass pollen and HDM SLIT tablets that are now available.

Given all the heterogeneity in approach, the generally positive conclusions of the published systematic review hide the underlying heterogeneity between studies. There are two key considerations. Firstly, not all products or approaches may be equally effective or have equal safety records. Secondly, there may be specific subgroups of patients who respond better to different approaches. The published systematic reviews have struggled to deliver useful subgroup analyses, mainly due to heterogeneity in study endpoints. With the move to harmonize study endpoints, there is now an opportunity to generate meta-analyses with sufficient numbers of participants to look at specific subgroups so as to help to make evidence based treatment decisions. It will be important though to ensure that results are not biased by studies examining older products using less well optimal study designs. Our follow-on systematic review will also offer the opportunity to include evidence from the more recent, larger and generally better designed clinical trials.

### Implications for policy, practice and future research

Systematic overviews of the literature are increasingly being used to inform policy deliberations as they can provide a comprehensive overview of the evidence landscape in relation to an important area of enquiry. Our overview has done this indicating that there is now substantial evidence that AIT—particularly if administered through the SCIT and SLIT routes—can be effective in improving clinically important outcomes in patients with AR/ARC with an acceptable safety profile. The evidence base is far less convincing in children due to lack of high quality trials in this age group. Also from systematic reviews the evidence in seasonal disease due to pollen is more consistent than for perennial disease. Importantly, since the cut-point date for evaluation in systematic reviews a number of large, adequately powered studies provide convincing evidence for the efficacy of SLIT for perennial mite allergy.

This review demonstrates the need for an updated review of AIT therapy, particularly in relation to further studies of the comparative effectiveness of these treatment routes, the patients most likely to benefit and least likely to experience significant harm, and the cost-effectiveness of AIT. More insights are also needed on how the effectiveness, safety and cost-effectiveness of AIT compares with other treatment modalities commonly used in the treatment of AR/ARC. The follow-on systematic review of AIT for ARC will allow these and related questions to be answered with considerably more detail and through so doing offer the opportunity to draw out recommendations for clinical practice.

## Additional files

**Additional file 1.** Search strategy.

**Additional file 2: Table S1.** RCTs included within systematic reviews.
